# Automated inverse optimization facilitates lower doses to normal tissue in pancreatic stereotactic body radiotherapy

**DOI:** 10.1371/journal.pone.0191036

**Published:** 2018-01-19

**Authors:** Ivaylo B. Mihaylov, Eric A. Mellon, Raphael Yechieli, Lorraine Portelance

**Affiliations:** Department of Radiation Oncology, University of Miami,Miami, FL, United States of America; North Shore Long Island Jewish Health System, UNITED STATES

## Abstract

**Purpose:**

Inverse planning is trial-and-error iterative process. This work introduces a fully automated inverse optimization approach, where the treatment plan is closely tailored to the unique patient anatomy. The auto-optimization is applied to pancreatic stereotactic body radiotherapy (SBRT).

**Materials and methods:**

The automation is based on stepwise reduction of dose-volume histograms (DVHs). Five uniformly spaced points, from 1% to 70% of the organ at risk (OAR) volumes, are used. Doses to those DVH points are iteratively decreased through multiple optimization runs. With each optimization run the doses to the OARs are decreased, while the dose homogeneity over the target is increased. The iterative process is terminated when a pre-specified dose heterogeneity over the target is reached. Twelve pancreatic cases were retrospectively studied. Doses to the target, maximum doses to duodenum, bowel, stomach, and spinal cord were evaluated. In addition, mean doses to liver and kidneys were tallied. The auto-optimized plans were compared to the actual treatment plans, which are based on national protocols.

**Results:**

The prescription dose to 95% of the planning target volume (PTV) is the same for the treatment and the auto-optimized plans. The average difference for maximum doses to duodenum, bowel, stomach, and spinal cord are -4.6 Gy, -1.8 Gy, -1.6 Gy, and -2.4 Gy respectively. The negative sign indicates lower doses with the auto-optimization. The average differences in the mean doses to liver and kidneys are -0.6 Gy, and -1.1 Gy to -1.5 Gy respectively.

**Conclusions:**

Automated inverse optimization holds great potential for personalization and tailoring of radiotherapy to particular patient anatomies. It can be utilized for normal tissue sparing or for an isotoxic dose escalation.

## Introduction

Despite the availability of modern multi-modal treatment options, pancreatic adenocarcinoma patients continue to have a very dismal 5-year survival prognosis of about 6% including for all stages combined.[1] Meanwhile, the rate of pancreatic cancer continues to increase at about 1.3% per year. Because of this trend, pancreatic cancer is expected to become the second leading cause of cancer death in not that distant future.[2]

The growing role of radiotherapy in pancreatic adenocarcinoma and the time-intensive and challenging nature of planning for dose-escalated radiotherapy have led us to seek further enhancement in the dose optimization algorithms by introducing automation. Many different algorithms have been proposed for improvements of the IMRT optimization process.[3–16] Initial work proposed fast, efficient inverse planning through reduced constraint optimization, where one of the major goals was to achieve quick optimization solution.[7, 10, 12] Another class of fast automated IMRT solutions is based on parameters derived from “expert” plan libraries, pre-generated for various patient anatomies and different treatment sites.[4–6, 9, 13] A variation of this approach was applied to breast treatments. It included a selection of beam and collimator angles from a library of plans, combined with optimization which utilized two-stage process incorporating forward and inverse optimization.[17] A more comprehensive approach for automatic IMRT optimization used beam angle look-up from a “knowledge” database, while dose and dose-volumetric objectives were iteratively reduced in the subsequent inverse plan optimization.[15] More recently, an automated IMRT optimization with iterative reduction of hot and cold spots was developed by generation of additional IMRT optimization structures.[14] Another variation of treatment plan automation was realized through unsupervised machine learning, where a set of standardized beam angles was generated on the basis of prior experience derived from treatment plans for different anatomical locations of the targets.[18]

This work introduces a form of automated inverse optimization where optimal (or near optimal) intensity maps are generated automatically for each unique patient and their particular anatomy. It is based on step-wise reduction of the dose-volume (DVH) histograms. Doses to several (five in this work) DVH points for each organ at risk (OAR) are iteratively decreased through multiple optimization runs. With each optimization run the doses to the OAR DVHs are decreased, while the dose homogeneity over the target is increased. The iterative process is terminated when a pre-specified dose heterogeneity over the target is reached. Therefore, the treatment plans are tailored to each individual case, such that the radiation doses to healthy tissue are minimized as low as reasonably achievable, without defying the therapeutic intent by compromising target doses and coverage.

## Materials and methods

### Patient data

In this work twelve pancreatic cancer patients are retrospectively studied. The retrospective chart review was approved by the institutional review board (University of Miami IRB protocol number 20160960) and it did not require informed consent. For all studied patients there was no duodenal wall invasion and they were treated with SBRT in our institution between January 2014 and September 2016. Patients received 6–8 cycles of chemotherapy, followed by radiotherapy, and finally by surgery if eligible. None of those patients had prior radiation therapy to the abdominal region.

For each case, gross tumor volume (GTV) was outlined on the planning CT. A planning target volume (PTV) was generated by expansion of the GTV ranging from 0.3 cm to 0.5 cm. In addition, duodenum, bowel, spinal cord, stomach, liver, and kidneys were outlined. All of those structures were used as dose-limiting organs at risk (OARs) in the inverse plan optimization and the clinical decision making of plan quality. Standardized dose prescription was used in all cases: 35 Gy to 95% of the PTV in five fractions. All patients were treated with volumetric modulated arc therapy (VMAT). The treatment plans used 3 to 6 full arcs, depending on patient anatomy. The details of the optimization objectives used for clinical planning are presented in **Table 1**. Treatment planning was performed with Varian’s Eclipse treatment planning system (TPS), utilizing its RapidArc capability.

**Table 1:** Dosimtric objectives used for clinical treatment plans.

	PTV	Duodenum	Bowel	Stomach	Cord	Liver	Kidneys
**Dose** **[Gy]**	35	30	30	30	20	10	10
**Volume/Fractional Volume**	95%	2 cc	2 cc	2 cc	Max dose	Mean dose	Mean dose

### Optimization automation

The proposed automated optimization approach is based on a dose-volume reduction scheme because dose-volume optimization is most widely used in modern radiotherapy. However, the automated optimization can also be used with other metrics such as generalized equivalent uniform doses,[19–21] dose-mass optimization,[22–25] or global energy minimization.[26]

In this work the automated optimization is applied with DVH objectives of the form

$$F^{j}=w^{j}\sum_{i\in V}{\left(\frac{d_{i}-d^{j}}{d^{j}}\right)}^{2}\Delta v_{i}$$ (1)

presented in **Eq 1**. *F*^*j*^ is the objective value, where: *w*^*j*^ is a user assigned weight, *V* denotes the volume of the anatomical structure of interest where the dose *d*_*i*_ is larger than the dose *d*^*j*^, *d*_*i*_ is the dose in voxel *i* of the volume *V*, *d*^*j*^ is the desired (objective) dose, and *v*_*i*_ is the normalized voxel volume with respect to the total organ volume *V*.[24, 27, 28] An optimization function of the form presented in **Eq 1** is necessary for each DVH optimization objective.

**Fig 1:**
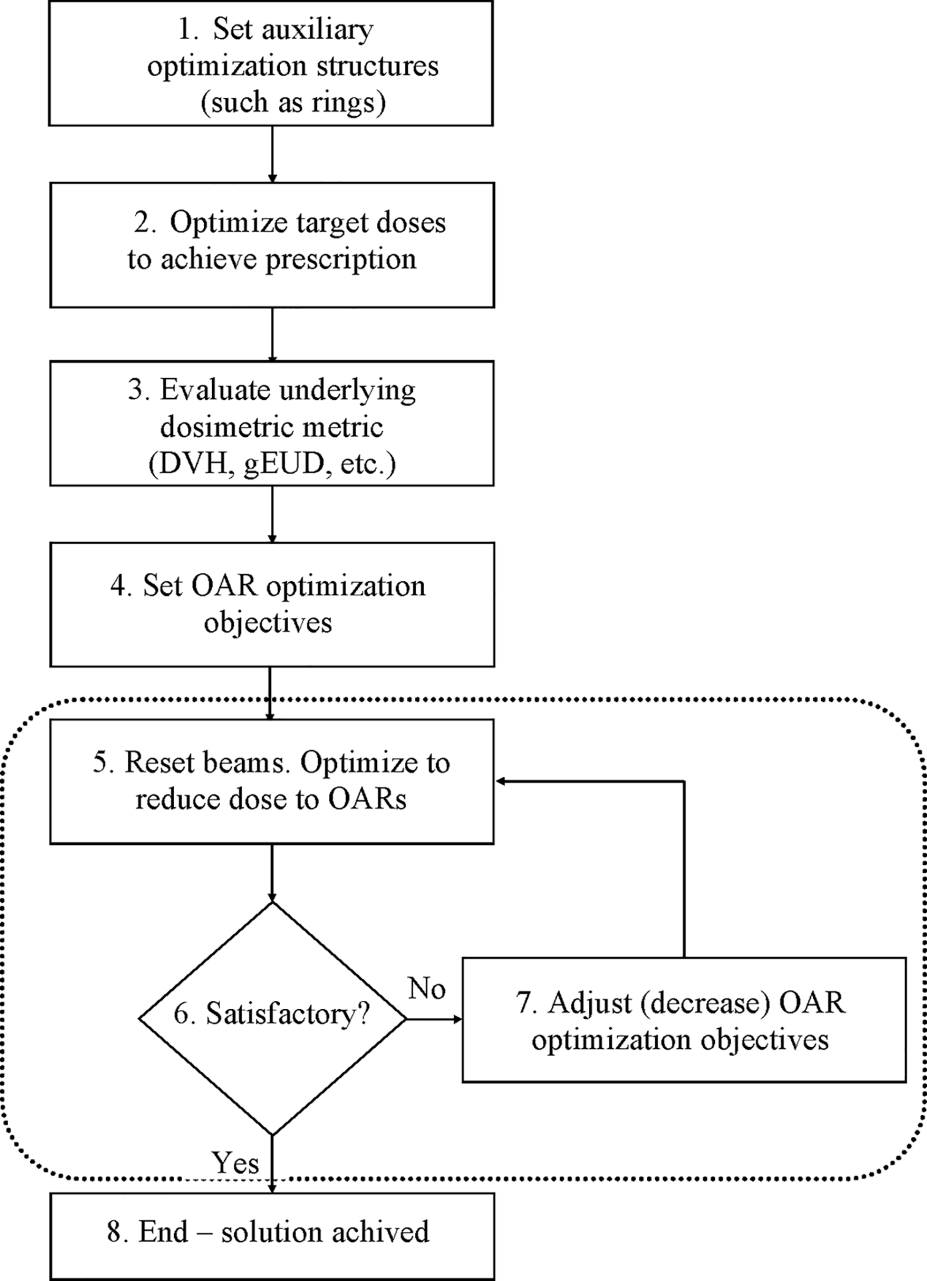
Flowchart of the automated inverse optimization. All of the steps presented in the figure are executed with a single button click. A prerequisite for the auto-optimization is that contours of the anatomical structures are outlined, the beams/arcs are set, the dose grid is positioned, and the number of treatment fractions is specified. Step 1 sets the auxiliary ring structures which control the dose fall off. Step 2 creates a plan with only PTV and auxiliary structures objectives. Step 3 evaluates the initial DVHs. Step 4 generates the initial OAR objectives. Steps 5, 6, and 7 are executed multiple times such that at each successive repetition the doses in the DVH objectives are progressively reduced. This stepwise DVH reduction is terminated when standard deviation of the dose over PTV reaches 5% of the prescription dose–step 8.

The automated process includes multiple steps (**Fig 1**). In the first step, shells or rings around the targets are generated as auxiliary structures to shape the dose fall-off from the target. In this step the target and the supplementary structures objectives are also set. The target objectives include minimum, maximum, and uniform doses, while the auxiliary structures objectives consist of maximum and mean doses.

In the second step, optimization proceeds on the targets defined in the first step. The aim of this initial optimization is solely to achieve the prescription dose for the target(s). The optimization objectives to the target(s) and the auxiliary structures, set in the first step above, are not altered any further in the subsequent steps.

In the third step, after a suitable solution is found in the first step, the OAR objectives are set. The type and the number of the OAR objectives is read from a configuration file, created by the user upfront. In this step the underlying dosimetric metric (e.g. DVH, EUD) is evaluated for all anatomical structures of interest–targets and OARs alike (step 3 on **Fig 1**), thereby estimating the OAR objective values. As an example, in the case of DVH optimization a set of predetermined fractional volumes are used as objectives—5 equi-spaced points on the cumulative DVH curve, spanning the range from 1% to 70% of the OAR volume of interest. OAR objective doses *d*^*j*^ from these initial DVH points are used as the basis for the determination of the OAR dose-volume objectives for the next step in the auto-optimization process.

In step 4, and again later in step 7, The OAR dose-volume objectives *d*^*j*^ are adjusted such that the OAR optimization objective values *F*^*j*^ (cf. **Eq 1**) are slightly larger (by ~5%) than the largest objective value for the targets. The adjustment of the OAR objective value is achieved by varying the dose for each preset fractional volume. Usually one of the target objectives (most often the dose uniformity) has the largest objective value, and it is the one used as the target objective according to which the OAR objectives are scaled. From experience we have determined that the scaling of the OAR doses in such a manner (about 5% larger than the target objective value) will properly “guide” the solution toward achieving the final goal, namely adequate target coverage for as low as reasonably achievable OAR sparing. This experience is derived from Pinnacle TPS (see below), but the actual value might be different for other TPSs and need to be evaluated by the user.

After setting the OAR doses for the predetermined metrics (step 4 in **Fig 1**) the optimization is performed again, but this time with both target and OAR optimization objectives in place (step 5). At the end of the next step, the solution is checked whether it satisfies some acceptance criterion (step 6). In the work presented here the acceptance criterion is that standard deviation of the dose across the PTV is less than 5%.[29] It should be pointed out, that the solution achieved by steps 1 and 2 result in target doses with high homogeneity and very low standard deviation of PTV dose (~ 1% to 2%). If the acceptance condition (step 6) is not satisfied, the desired doses *d*^*j*^ to the OARs (for each fractional volume) are lowered again such that all OAR objectives *F*^*j*^ become about 5% larger than the largest target objective value (step 7). The entire optimization is carried again (step 5). The loop (denoted by the dashed rectangle on the figure) proceeds through steps 5, 6, and 7 until the acceptance condition in step 6 is achieved. At that point the auto-optimization is terminated–step 8. The successive reduction of the OAR objective doses *d*^*j*^ in the loop over steps 5, 6, and 7 will gradually increase the dose heterogeneity over the PTV until the pre-specified optimization termination condition is met in step 6.

The process depicted on **Fig 1** is in essence a stepwise reduction of the OAR optimization doses *d*^*j*^ (or equivalently stepwise increase of the OAR objective function values *F*^*j*^). The result of this stepwise dose decrease is a gradual convergence of the optimization toward a balanced solution, where therapeutic doses are delivered to the tumors, while at the same time sparing the OARs as much as reasonably achievable.

In routine clinical practice inverse optimization is limited to no more than 2 or 3 objective functions per OAR. However, in the presented automated inverse optimization there might be an arbitrary number of objective functions for each. In this investigation 5 individual DVH objectives were used per OAR. Those were set for equi-spaced relative OAR fractional volumes between 1% (high dose) and 70% (low dose). In order to increase the flexibility of the auto-optimization scheme, the OAR importance and/or the importance of a single dose-volume (such as maximum 1%) objective can be pre-specified by the user on a sliding scale.

The auto-optimization platform is developed as a plugin to a research version of a commercially available TPS (Pinnacle, Philips Radiation Oncology Solutions, Fitchburg, Wisconsin). Note that each of the steps 1 and 5 on **Fig 1** are realized as one Pinnacle optimization cycle. This cycle is terminated either by reaching 100 iterations, or by a change in the composite objective function (which is a sum of all *F*^*j*^ from **Eq 1** over all structures) of less than 10^−4^. Every time step 5 (cf. **Fig 1**) is executed the beams are reset automatically through Pinnacle’s built in “Reset Beams” functionality.

### Setup for pancreatic cancer planning comparison

For each of the twelve cases an automated VMAT plan was generated with the auto-optimization module described above. The auto-optimized plans used the same initial parameters as the original plans such as dose grid size and resolution, number of full dynamic arcs, and photon energies. The prescription was set such that 95% of the PTV volume received the prescription dose of 35 Gy. Three rings were used for control of the dose fall-off. Each of the rings was 1 cm in size and they were at 0.5, 3.5, and 5.5 cm from the PTV. The PTV optimization objectives (step 2 in **Fig 1**) were set to minimum, maximum, and uniform doses with relative weights *w*^*j*^ of 100. Two objectives for each of the three rings were specified in terms of maximum and mean doses. The relative weight *w*^*j*^ for the rings was set to 80. In step 5 of the auto-optimization five objectives were set for each OAR for equi-spaced fractional volumes ranging from 1% to 70%. The relative objective weights *w*^*j*^ for all OARs were set to one. The loop denoted by the dotted line on **Fig 1** was allowed to run (steps 5, 6, and 7) until the standard deviation of the PTV dose became approximately 5% of the prescription dose. In each case two to three cycles of steps 5, 6, and 7 were sufficient for the termination condition to be achieved.

### Analyses

The analyses of the obtained result were based on clinically used end points. They included doses to 95% and 5% of the PTV, as well as doses to 2% (surrogates for maximum doses) of duodenum, spinal cord, bowel, and stomach.[30] Mean doses to liver and kidneys were also interrogated. All these quantities, termed dose indices (DIs) hereafter, were normalized for better visualization.[19, 31–34] The normalization was performed with respect to the DIs derived from the treatment plans. If a normalized DI is greater than unity, then the absolute dose for that particular patient and index, resulting from the auto-optimized plan is larger, and vice versa.

In addition to direct comparison of the DIs non-parametric statistical tests were performed on the absolute DIs. The statistical significance was established with related samples Wilcoxon singed rank test.

## Results

**Fig 2:**
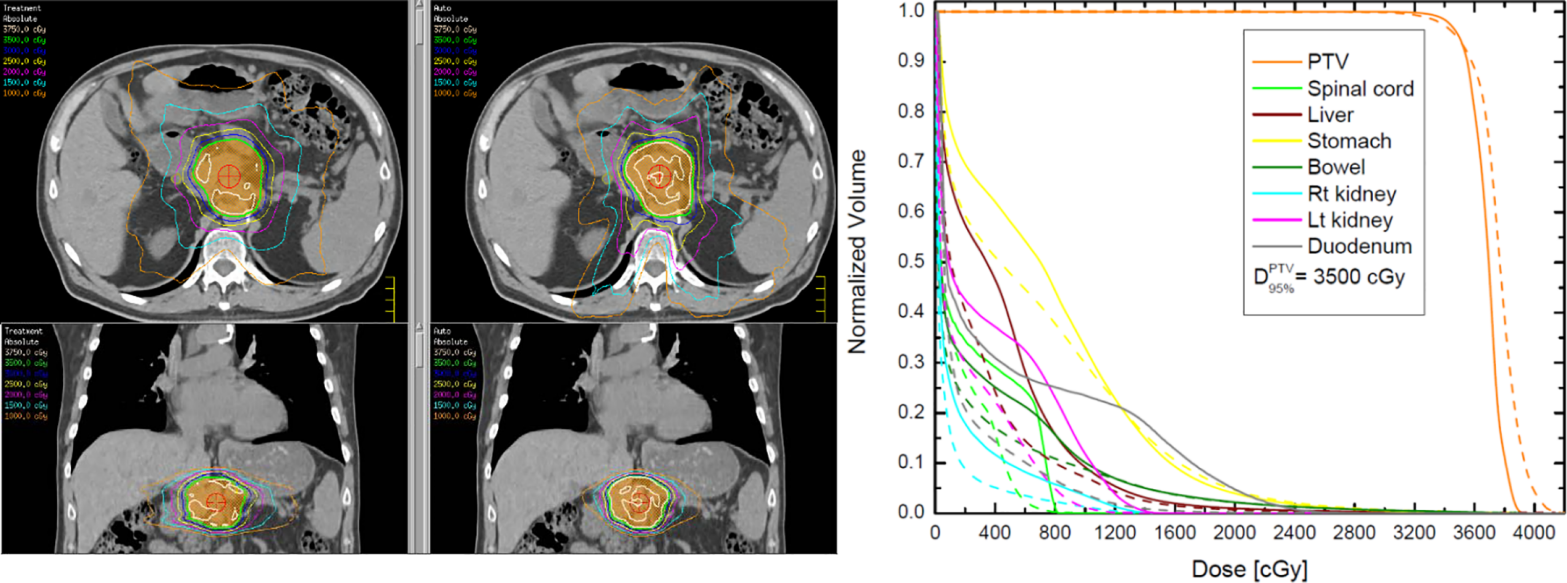
Comparison of isodose plots (left) and DVHs between a manually planned treatment and an auto-optimized plan. The left column displays the absolute doses of the treatment plan on axial and coronal views, while the right column corresponds displays the auto-plan for the same patient. The colorwash structure is the PTV, while the green isodose line is the prescription dose of 35 Gy. The DVH plot on the right outlines the treatment DVHs (solid lines) and the auto-optimized DVHs (dashed lines).

**Fig 2** demonstrates isodose plots and DVHs for one case from the patient cohort. There are two columns in the isodose plots (left panel of the figure), where the left column corresponds to the treatment isodoses, while the right column depicts the auto-optimized isodoses. It is evident from the plots that the prescription isodose (green) conforms to the PTV (orange) well in both plans. The solid lines on the right outline the DVHs from the treatment plans, while the dashed DVHs correspond to the auto-plan. The differences in the maximum dose of the duodenum, the bowel, the stomach and the spinal cord were -35%, -5%, -6%, and -27% respectively. The differences in the mean doses to liver, the left, and the right kidneys were -26%, -48%, and -46% respectively. The negative values indicate lower absolute doses from the auto-optimized VMAT plan.

**Fig 3:**
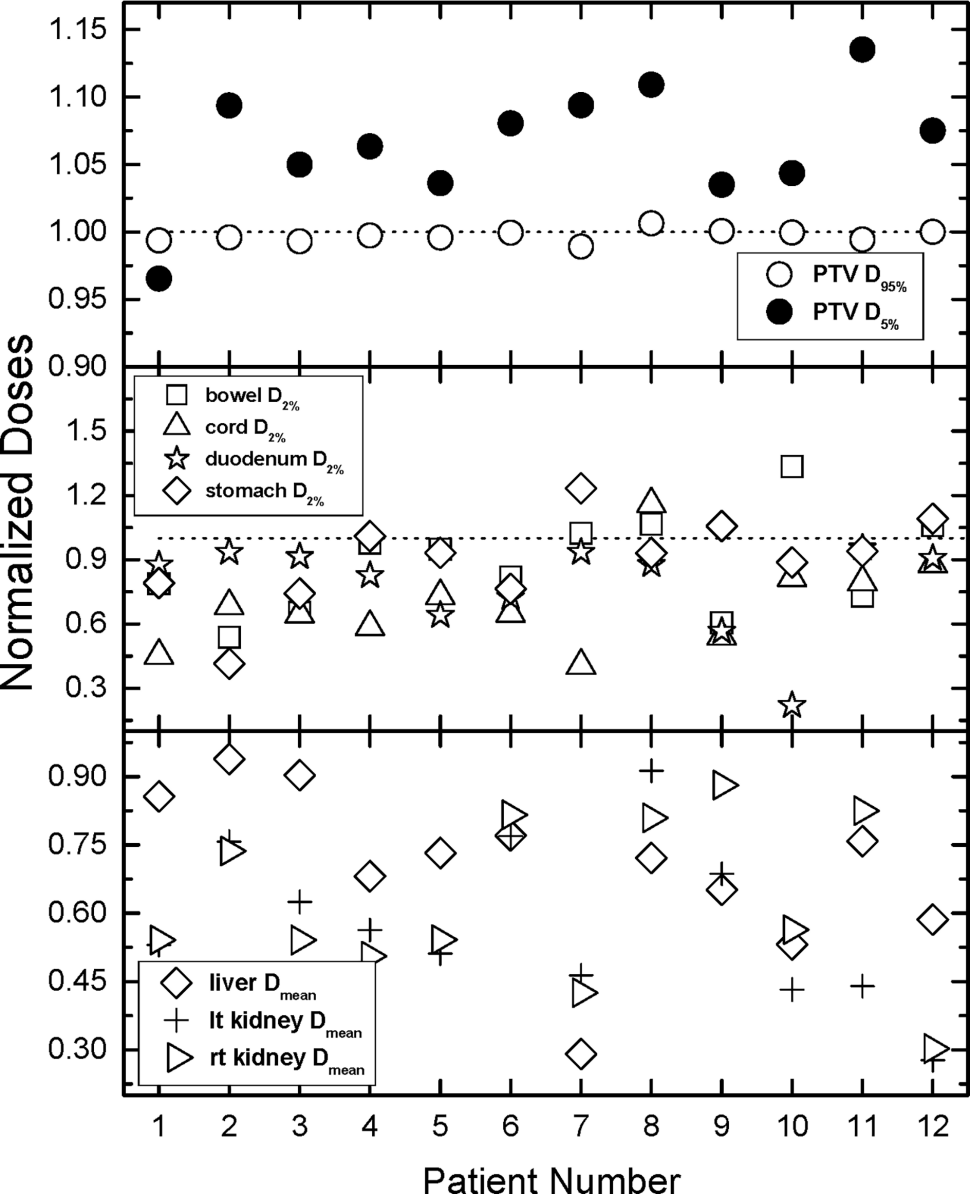
Comparison of all normalized tallied indices. Normalization is performed with respect to the doses derived from the treatment plans and plotted as a ratio of auto-plan over manual treatment plan. Values greater than one indicate more dose for the auto-plan compared to the manual plan, values less than one indicate less dose for the auto-plan, and values equal to one indicate equal doses for the two compared plans. The top panel outlines the normalized doses to 95% and 5% of the PTV. The middle panel contains the data for doses to 2% of duodenum, bowel, stomach, and spinal cord. 2% of the volume for those OARs have been used as surrogates for maximum doses. The bottom panel of the figure outlines the average doses to liver and kidneys. Note that while most normalized maximum doses in the middle panel are smaller (majority) than unity, all normalized mean doses in the bottom panel which are smaller than unity.

**Fig 3** presents the data for all tallied DIs. In addition to the DIs, unity (dashed line) is also plotted in the figure, indicating where the absolute doses from the treatment and the auto-plans are equal. From the panel is evident that 95% of the PTV receives the same dose with either optimization approach, as intended by the prescription. The doses to 5% of the PTV with the auto-optimized plans are somewhat larger than the doses from the treatment plans by 6% on average (range -3.5% to 13.5%). The middle panel outlines the data for D2% to the stomach, the bowel, the spinal cord, and the duodenum. The differences range from -78% (duodenum patient 10) to -4.6% (patient 11), with average difference of -21%. The situation with spinal cord maximum doses is similar where the average difference is -32% with a range from -59% to 10%. The average differences in bowel and stomach are -5% (-34% to 33%) and -13% (-50% to 23%). The differences in the maximum doses to bowel and stomach vary more than in the case of duodenum, but this is a result of the higher priority given to the duodenum sparing in the auto-optimization. Nonetheless, the results indicate that the majority of the tallied maximum doses are lower with auto-optimization. The bottom panel of **Fig 3** presents the mean doses to liver and kidneys. The scale of the plot indicates that all mean doses are lower in the auto-optimized plans, since the normalized DIs span the range from 0.3 to 0.95.

**Table 2** presents the average absolute doses for all tallied OAR DIs, placing in perspective the relative differences from **Fig 3**. On the top row the actual treatment doses (averaged over the patient cohort) are presented, while on the bottom row the average doses from the auto-optimized plans are outlined. In addition to the average doses, in the parentheses the range of that DI is also given. The table clearly indicates that the duodenum receives the highest maximum doses followed by stomach, bowel, and spinal cord. Furthermore, the most clinically significant dose reductions are those achieved for duodenum, bowel, and stomach since they are in close proximity to the target.

**Table 2:** Average absolute doses to all OARs for the tallied dose indices. The last row indicates the *p*-values derived from related samples Wilcoxon singed rank statistical test.

	Duodenum D_2%_ [Gy]	Bowel D_2%_ [Gy]	Stomach D_2%_ [Gy]	Cord D_2%_ [Gy]	Liver D_mean_ [Gy]	Lt kidney D_mean_ [Gy]	Rt Kidney D_mean_ [Gy]
**Average Value Treatment** **(range)**	24.7 (14.9–31.7)	16.0 (7.9–20.1)	17.6 (2.0–30.0)	7.3 (4.0–11.6)	2.1 (0.5–4.3)	2.5 (1.2–4.9)	3.3 (0.2–8.5)
**Average Value Auto** **(range)**	20.1 (3.3–29.7)	14.2 (6.5–19.9)	16.0 (2.0–28.3)	4.9 (2.5–8.4)	1.5 (0.5–3.4)	1.4 (0.5–2.3)	1.9 (0.2–7.0)
***p*-values**	<0.05	0.071	0.071	<0.05	<0.05	<0.05	<0.05

## Discussion

Each auto-optimized plan is specifically tailored to the individual patient anatomy. In contrast, the database look-up and expert plan utilization approaches are in effect population based methods, where the expert plan is as good as the specified planning objectives, or the experience of the planner. Notably, human anatomies are rather similar in many aspects, but rarely if never identical. Even if the relative anatomies are very similar tissue properties are likely to be different between patients. Therefore, the use of “expert” dosimetric objectives may be far from optimal on patient-by-patient basis.

The auto-optimization presented in this work creates plans on the basis of minimal assumptions. The optimization objectives are not based on any protocols, guidelines, or clinical practices. Instead, the doses are minimized as much as reasonably achievable, depending on sufficient target coverage and pre-set dose uniformity over the target. In addition, all of the OAR goals are set as optimization objectives rather than constraints. While the optimization objective is a desired goal, the optimization constraint is a “must do” restriction. The result is that if an objective goal is set as a constraint, the optimization algorithm would satisfy it first and will attempt to find a solution for the objectives by manipulating the remaining free parameters. As a result of that inflexibility a portion of the solution space may be blocked and thereby sub-optimal solutions will emerge. If on the other hand, only optimization objectives are utilized as in this work, the optimization algorithm would maintain its flexibility and it is very possible that a solution with better trade-off could be achieved. However, it is quite possible that the auto-optimization is unable to satisfy a clinical “must do: constraint. In this scenario the auto-plan solution may be used as a starting point, namely all of the dosimetric objectives calculated by the auto-planning are used, and only those which affect the achievement of the clinical constraints are manually adjusted in further optimization.

The presented results indicate that this auto-optimization approach is capable of reducing doses to nearby anatomical structures. Close inspection of **Fig 3** indicates that the farther away an OAR is from the target, the better sparing can be accomplished with the auto-optimization. Therefore, the auto-optimization can be used “as is” for dose reduction to nearby OARs, or it can be utilized for dose escalation. **Fig 3** indicates that for patients 1, 2, 3, 6, and 11 an approximately 15% increase of the prescription dose would lead to almost isotoxic dose escalation where the auto-optimized doses can be “boosted” to 8 Gy per fraction for a total dose of 40 Gy. This dose escalation may be used to overcome the known radio-resistance of pancreatic cancers. [35] [36, 37] Therefore, the dose escalation allowed by the auto-optimization scheme may be potentially translated into higher resection rates and improved overall survival. The presented results suggest that this might be an option in about 40% (5 out of 12) of the cases which require radiotherapy.

The presented auto-optimization scheme is not geared toward quick solution. It is aimed in “pushing” the limits as much as possible to achieve low doses to the healthy anatomical structures in target vicinity. Although not rigorously benchmarked, the optimization needs a couple of hours to find a solution. This may not be optimal in real time, but it is perfectly suitable to be used overnight when the computers are unutilized. Nonetheless, the auto-optimization saves tremendous amount of time for the planners. As it was mentioned above, there is virtually no limit on how many DVH points are used as objectives and they are automatically adjusted during the optimization process. This is unachievable for a planner since this will require prohibitively long time.

## Conclusion

This work outlined a new inverse optimization approach, based on automated reduction of dose-volume objectives. It is tailored to each individual patient case and is based on minimum assumptions. Virtually, all that is required are the prescription goals and the desirable dose homogeneity over the target. This auto-optimization scheme was applied to pancreatic cancer treated with SBRT. It was demonstrated that in some cases auto-optimization is capable of reducing doses to normal tissue in comparison to treatment plans created by senior very experienced dosimetrists. The available dose reduction can be utilized “as is” for reduction of the complications rates, or it can be used for isotoxic dose escalation in the prescription, which might be an important factor in the management of the radio-resistant pancreatic cancer. Since the presented auto-optimization approach requires very minimal input and virtually no supervision it can be performed in background and/or after hours and can be used as a starting point or for comparison to conventional treatment plans. It may help less experienced users achieve better quality radiotherapy treatment plans.

## Supporting information

S1 Dataset FileData.zip contains the absolute dosimetric indices (DIs) for the organs at risk (OARs) used in the optimization process. The content of the file is patient number, dose from the treatment plan, dose from the automated-inverse optimization plan. The file names in the FileData.zip indicate the type of the DI (e.g. bowel.0.02 indicates that this is doze to 2% of the bowel).
(ZIP)
